# Screening and Treatment for Co-occurring Gambling and Substance Use: A Scoping Review

**DOI:** 10.1007/s10899-023-10240-z

**Published:** 2023-07-26

**Authors:** Elisabeth Yarbakhsh, Anke van der Sterren, Devin Bowles

**Affiliations:** 1 Alcohol, Tobacco and Other Drug Association, ACT (ATODA), Canberra, Australia; 2 College of Arts and Social Sciences, Australian National University, Canberra, Australia; 3 School of Population Health, University of New South Wales, Sydney, Australia; 4 ACT Council of Social Service (ACTCOSS), Canberra, Australia

**Keywords:** Gambling, Substance use, Screening, Treatment, Co-morbidity

## Abstract

There is a high prevalence of gambling harms co-occurring with substance use harms. Where harms are co-occurring, they may be experienced as more severe. However, there is little evidence that services are systematically screening for such co-occurring harms in treatment-seeking populations. Furthermore, treatment modalities remain relatively under-developed, with treatment usually addressing only one source of harm.

This scoping review looks at the current literature on screening and therapeutic interventions for co-occurring gambling and substance use harms to understand how co-occurring harms may be managed in a treatment setting. It draws together available data on the intersections of substance use harms and gambling related harms, in a treatment context.

This research identifies a range of potentially useful validated tools for clinicians in substance use treatment settings to screen for gambling harms. For workers in gambling treatment settings who are seeking validated tools to screen for co-occurring substance use harms, the literature provides less guidance.

The validated toolbox of therapeutic interventions for those experiencing co-occurring substance use and gambling harms is relatively sparse. Psychosocial interventions appear to offer the best outcomes on gambling measures for those experiencing co-occurring substance use harms. Further research is needed to establish the benefits of different combinations of treatment and treatment types in achieving reductions across both substance use and gambling harms, when these harms are experienced concurrently.

## Introduction

Both gambling and substance use have a range of consequences that may, for a subset of the population, be experienced as harms (Kourgiantakis et al., 2021). The harms associated with gambling and substance use can be severe and tend to be cascading, such that one form of harm can precipitate and exacerbate other forms of harm (Eby et al., 2016; Algren et al., 2018; Langham et al., 2016; Loxley et al., 2004). Harms are not evenly distributed or experienced across the population, and individuals experience different risk factors that may make them more or less likely to experience harms. Experiencing harms from substance use can increase the risk of experiencing gambling harms and vice versa (Grant & Chamberlain, 2014). The co-occurrence of substance use harms and gambling harms is well established in the literature (Cowlishaw et al., 2014; Johansson et al., 2009). A systematic review and meta-analysis of available population surveys found that 57.5% of individuals experiencing gambling harms experienced co-occurring substance use harms (Lorains et al., 2011). Individuals who have a lifetime experience of gambling harms are up to 7 times more likely to experience substance use harms than non-gamblers or those assessed as ‘recreational gamblers’ (Grant & Chamberlain, 2020). Conversely, those with a lifetime experience of substance use harms may be as much as 10 times more likely to experience gambling harms than the general population (Potenza et al., 2002).

Co-occurring substance use harms and gambling harms can be a complicating factor in treatment and can result in poorer treatment outcomes (Wieczorek & Dąbrowska, 2023; Dowling et al., 2015). Individuals experiencing both substance use harms and gambling harms are more likely to cease treatment prematurely (Milton et al., 2020). Some studies have found evidence of ‘addiction substitution’ whereby successful treatment for one form of harm may, in particularly vulnerable populations, precipitate other potentially harmful behaviors (Kim et al., 2021). Offering holistic screening and therapeutic interventions that capture harms from both substance use and gambling can be an efficient and cost-effective way to reach a high-risk population and to reduce net harms (Christo et al., 2003). However, there is little evidence that a holistic approach is currently used in addressing such co-occurring harms (Sherba & Martt, 2015; Wolinski et al., 2003; ATODA, 2022). A 2020 Australian study that addressed the capacity of mental health clinicians (including those working in alcohol, tobacco and other drug [ATOD] services) to respond to gambling harms, found that most had ‘limited knowledge of screening tools to detect PG [problem gambling]’ (Manning et al., 2020). In the same study, only 16% of clinicians were found to screen ‘often’ or ‘always’ and few expressed confidence in their ability to provide appropriate therapeutic interventions for gambling harms. Only 12.5% reported receiving training in gambling harms, and those that had, displayed higher levels of knowledge about gambling (in the context of mental illness), more positive attitudes about responding to gambling issues, and more confidence in screening for or detecting gambling harms (Manning et al., 2020). Although this study was not specific to the ATOD sector, the broad findings around inadequate screening and low confidence in ability to respond to gambling harms are likely to be mirrored in the ATOD workforce. In the past, very few specialist ATOD treatment services have provided gambling treatment to service users (Wolinski et al., 2003). ATOD workers have identified gambling as a significant training gap (ATODA, 2022). In a qualitative study of ATOD service users in Ohio, almost two-thirds of participants reported gambling in the 6 months prior (Sherba & Martt, 2015). Of these participants, only 22.2% reported ever having been asked about gambling while receiving ATOD treatment services and just 12.5% reported ever having had gambling treatment services offered to them.

This review seeks to understand how co-occurring substance use and gambling harms are being managed in treatment settings. It draws together available data on the intersections of substance use harms and gambling related harms in a treatment context with the aim of establishing an evidence base on which services can begin to build best practice approaches for responding to such co-occurring harms.

## Methods

In January 2023, the authors completed a scoping review of the available literature on screening and therapeutic interventions in respect of co-occurring substance use and gambling harms. The review formed the first phase of a broader project aiming to identify and support services to implement best practice approaches in responding to co-occurring substance use and gambling harms. As such, the review was intended to scope for:

- validated screening tools for co-occurring substance use harms and gambling harms; and.
- current practice in therapeutic responses for co-occurring substance use harms and gambling harms.

This process would allow the authors to develop a broad understanding of how services might appropriately respond to the presentation of co-occurring substance use harms and gambling harms. A scoping review was undertaken to collate data where the available evidence and parameters of the field remained largely unknown and unexplored (Munn et al., 2018).

**Fig. 1:**
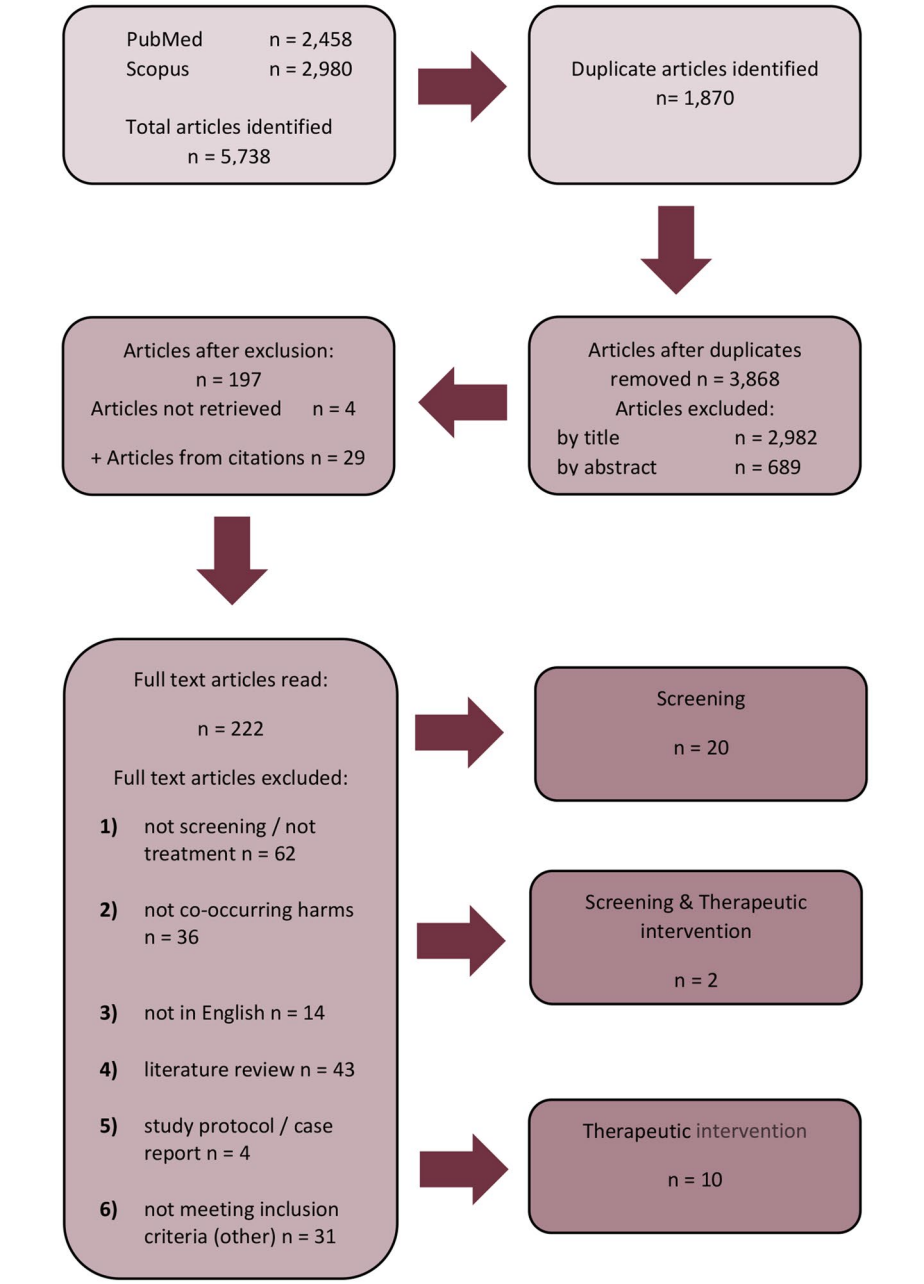
Scoping review method (PRISMA-ScR chart)

In undertaking this study, the authors utilized a method modified from the Preferred Reporting Items for Systematic Reviews and Meta-Analysis Protocols for Scoping Reviews (PRISMA-ScR). This review draws on the guidelines for conducting systematic scoping reviews developed by the Joanna Briggs Institute (Peters et al., 2015, 2020; Tricco et al., 2018). The method broadly involves three stages: identification of records; screening of records; and inclusion of records for data extraction. The steps undertaken across these three stages are outlined in Fig. 1.

### Identification

A search strategy was developed with advice from an academic research consultant at a university library. Two academic databases were chosen: PubMed and Scopus. The use of only two databases is a limitation of the study that may be addressed in future research. The PubMed search strategy is shown in Table 1.

**Table 1:** PubMed Search Strategy

	*AND*					
	*OR*				*OR*	
*PubMed (primary search strategy)*	(((“alcohol and drug*”) OR (“alcohol and other drug*”) OR (“drug and alcohol)) OR (“drugs and alcohol”)*”*	*“Drug Users”*	*“Substance-Related Disorders”*	*Substance Abuse Treatment”*	*“Gambling Disorders”*	*“Gambling”*

Results from the search of databases included literature spanning the disciplines of healthcare, neuroscience, psychiatry, psychology, social work and other social sciences. Results were exported into EndNote and duplicates were removed using the protocol developed by Bramer et al. (2016). In addition to records removed in EndNote, the authors detected and removed 17 duplicate records during the review process.

It was anticipated by the authors that reports, practice guidelines and treatment manuals would provide a rich source of supplementary data. To capture this grey literature a secondary search of Google was undertaken. Results were examined systematically up to, and including, page 30 (300 results generated), as recommended by Haddaway et al. (2015; Piasecki et al., 2018). Where links led to academic articles, these were automatically exported into Endnote; where links were directed to organisational or government websites, a further search was conducted of publications appearing on these sites. Searches were restricted to articles published after December 2001.

### Screening

Inclusion and exclusion criteria were developed by the authors and applied to the articles that remained after de-duplication. Screening involved three stages (a) screening of titles; (b) screening of abstracts; and (c) screening of full-text articles. Articles included for data extraction met the criteria of being in English and about screening and/or therapeutic interventions for gambling and/or substance use harms where such harms were identified as co-occurring. Articles were excluded where the substance use harm referred primarily to tobacco smoking or vaping; where the research was wholly or primarily an animal study; where the focus of the study was on Parkinson’s disease; or where the authors were concerned with the neurobiological underpinnings of addiction. Letters to the editor, editorials (including introductions to special issues) and other forms of commentary were excluded. After identifying relevant cited material, reviews were excluded except where they offered additional analysis.

All papers identified during the screening process and included as eligible for data extraction were grouped into one of three categories: screening; therapeutic intervention; and both screening and therapeutic intervention.

### Inclusion for Data Extraction

The response to co-occurring harms is considered, in this review, to be a two-phase process involving (1) screening and (2) therapeutic intervention. Screening allows a clinician to identify individuals experiencing (or at risk of experiencing) substance use or gambling harms and, in some cases, to assess the degree of harm being experienced. Screening provides a necessary first step prior to in-depth diagnostic assessment and therapeutic intervention. Therapeutic interventions are varied and can include both on-referral or treatment in the service setting.

To scope current practice in screening of co-occurring substance-use and gambling harms, the authors looked at the range of validated tools that have been used to screen or otherwise assess for the following:

- gambling/gambling harms in an ATOD treatment setting;
- substance use/substance-use harms in a gambling treatment setting;
- both gambling/gambling harms and substance use/substance use harms in a treatment setting, amongst a treatment-seeking population (including primary health care) or in the general population.

To scope current practice in therapeutic interventions for co-occurring substance-use and gambling harms, the authors looked at validated tools or working models that offered possible treatment or other intervention for the following:

- gambling treatment (or other therapeutic intervention) where there was co-occurring substance use harm;
- substance use treatment (or other therapeutic intervention) where there was co-occurring gambling harm;
- treatment (or other therapeutic intervention) that targeted both gambling and substance use, where harms were co-occurring.

## Results

A search of the databases and search engine identified 3,868 unique articles (following de-duplication). After screening, 32 articles were included for extraction. These referred to either screening (n = 20); therapeutic intervention (n = 10); or both screening and therapeutic intervention (n = 2).

### Screening

Across the dataset, 19 screening tools were identified (see Table 2). These tools screened for:

- gambling in substance-use treatment settings or, in other treatment settings where there was a significant clinical caseload of substance-use treatment needs (n = 11).
- both gambling and substance use and were validated across a range of settings and/or populations (n = 4).
- underlying behavioral traits or risk factors in the general population (n = 4).

**Table 2:** Screening tools

Tool	Items	Purpose	Setting	Study	Outcome
4Es	40 items	Predict gambling risk based on factors of Escape, Esteem, Excess and Excitement; distinguish gambling risk from alcohol harms	Non-treatment seeking	Rockloff and Dyer, 2006	In sample of 2,577 (Study 2): Escape, excitement and excess independently predict PGSI gambling problems. Escape and excess independently distinguish gambling from substance use.
The Alfred Screening Tool for Problem Gambling	4 items	Screen for problem gambling (self or other) in mental health service	Mental health	de Castella, 2011	Identified gambling harms in mental health treatment seeking population
ASI-G	5 items	Assess severity of gambling problems	Gambling treatment; opioid use treatment	Petry, 2003	In sample of 598: adequate to good Cronbach’s α (0.90); significant correlations between ASI-G scores and other indices (SOGS, DSM, TLFB)
BBGS	3 items	Screen for gambling disorder (DSM-5)	Methadone maintenance treatment (MMT) outpatient clinic	Himelhoch et al., 2015	In sample of 300: sensitivity (0.909); specificity (0.865); positive predictive value (0.821); negative predictive value (0.933)
			Substance use treatment	Rowe et al., 2015	Inclusion in treatment manual for ATOD workers
CHI-T	15 items	Measure transdiagnostic compulsivity	Non-treatment seeking	Chamberlain and Grant, 2018	The CHI-T had good convergent validity, with total scores correlating significantly with gambling disorder symptoms and with obsessive-compulsive symptoms. CHI-T total scores were also significantly elevated in participants who had a current substance use disorder versus those who did not.
CHAT	9 risk factors	Develop multi-item general practice tool	Primary health	Goodyear-Smith et al., 2004	In sample of 2,543 patients across 20 GP clinics, 2.8% self-assessed drug use harm, 3.2% gambling harm and 10.8% alcohol harm.
		Screen for health risk factors for follow-up in primary care		Goodyear-Smith et al., 2009	In sample of 755: alcohol: sensitivity 80%, specificity 85%; drug use: sensitivity 64%, specificity 98%; gambling: sensitivity 80%, specificity 98%.
		Testing of online eCHAT tool in primary care		Goodyear-Smith et al., 2013	In sample of 196 patients: 97% found iPad-based eCHAT easy to use; 9% had concerns about data privacy.
		Testing of CHAT tool in veteran health care		Goodyear-Smith et al., 2021	In a sample of 34: VeCHAT tool proved acceptable to veterans and Veterans’ Affairs staff
		Feasibility study of the CHAT		Elley et al., 2014	In sample of 107: CHAT found to be acceptable. 2 objections to alcohol questions; no other objections recorded.
EIGHT	8 items	Screen for pathological gambling or problem gambling (DSM-IV)	Substance use treatment	Sullivan, 2007	In sample of 676 in AOD setting: high correlation with SOGS (83.9%)
GDSQ-P	27 items	Screen for gambling disorder (DSM-5)	Substance use treatment (residential)	Maarefvand et al., 2019	In sample of 503: high sensitivity (0.99); specificity (0.98) and accuracy (0.98)
ICB	33 items	Measure impulsive-compulsive behaviors	Non-treatment seeking	Guo et al., 2017	In sample of 687: Impulsive-Compulsions and Compulsive-Impulsions yielded very good Cronbach’s α of 0.89 and 0.84 respectively. The ICB Checklist is best utilised by examining each item endorsed and corresponding severity rating. The total sum of each subscale may indicate whether there is a stronger inclination towards impulsive or compulsive behaviors.
Lie/Bet	2 items	Screen for pathological gambling (DSM-IV)	MMT outpatient clinic	Himelhoch et al., 2015	In sample of 300: sensitivity (0.942); specificity (0.657); positive predictive value (0.651); negative predictive value (0.944)
mASI	27 items (approx. 1 h completion)	Secondary screen or assess for gambling problems and substance use problems	Substance use/ non-substance use treatment (outpatient)	Denis et al., 2016	In sample of 833: The Cronbach’s α ranged from 0.63 to 0.87 and could be considered good for medical, alcohol, and gambling domains; acceptable for employment/ support, drug, tobacco, and psychiatric domains; and questionable for legal and family /social domains.
NODS	17 items	Screen for risky gambling, harmful gambling, and pathological gambling (DSM-IV)	Substance use treatment	Wickwire et al., 2008	In sample of 157: good Cronbach’s α (0.88); positive correlation with SOGS (r = .85, p < .001)
NODS-CLiP	3 items	Screen for Loss of Control + Lying + Preoccupation items of NODS	MMT outpatient clinic	Himelhoch et al., 2015	In sample of 300: sensitivity (1); specificity (0.539); positive predictive value (0.596); negative predictive value (1)
			Individuals recruited through substance use settings	Volberg et al., 2011	In sample of 375: 3-item NODS-CPR had higher diagnostic efficiency (90.1% compared to 86.4% for NODS-CLiP)
NODS-PERC	4 items	Screen for Preoccupation + Escape + Risked Relationships + Chasing items of NODS	MMT outpatient clinic	Himelhoch et al., 2015	In sample of 300: sensitivity (1); specificity (0.573); positive predictive value (0.614); negative predictive value (1)
PGSI	9 items	Screen non-problem gambler, low-risk gambler, moderate-risk gambler, problem gambler (self-assess)	Substance use treatment	Rowe et al., 2015	Inclusion in treatment manual for ATOD workers
SPQ	160 questions (10 × 16 domains)	Assess addictive behaviors	Addiction treatment (residential)	Christo et al., 2003	In sample of 497 (clinical)/ 508 (non-clinical): SPQ alcohol had high correlation with CAGE, SADQ, SMAST; SPQ recreation drugs scale had strong correlation with SODQ and SDS; SPQ prescription drugs had correlation with 6/8 validated scales; SPQ gambling had strong correlation with SOGS and correlation with 2 validated drug use scales. Cronbach’s α coefficient was high (between 0.82 and 0.98) across subscales.
			University students	MacLaren and Best, 2010	In sample of 948 university students: adequate inter-item reliability using Cronbach’s α (between 0.81 and 0.96).
SSBA	40 questions (4 × 10 domains)	Screen for addiction problems	Non-treatment seeking	Schluter et al., 2018	In sample of 6,000: AUC values were moderate-to-high demonstrating overall ability of each subscale to discriminate between individuals who did/ did not self-report problematic engagement in a target behavior
SSOGS	7 items	Screen for pathological gambling (DSM-III)	Substance use treatment (residential)	Nelson and Oehlert, 2008	In sample of 316: the SOGS internal consistency (coefficient α) for all items was 0.91. The 7-item SSOGS data yielded an internal consistency of 0.79.
Vices’ questionnaire	44 questions (22 × 2)	Screen for drug and addictive behaviors (‘vices’); distinguish ‘wanting’ from ‘liking’	Non-treatment seeking	Dale et al., 2016	In sample of 479: the ‘liking’/’wanting’ version of the survey had higher reliability and validity than the simpler ‘desire’ assessment.

There were no tools identified in the literature that screened specifically for substance use in a gambling treatment setting. There were 11 gambling screening tools that had been validated in substance use treatment settings, including in mental health treatment settings where substance use affects a significant proportion of service users (de Castella, 2011; Himelhoch et al., 2015; Maarefvand et al., 2019; Nelson and Oehlert, 2008; Petry, 2003; Rowe et al., 2015; Sullivan, 2007; Volberg et al., 2011; and Wickwire et al., 2008). Four broad spectrum or multi-diagnostic screening tools were identified. These tools screened for both substance use and gambling in either a treatment setting (Christo et al., 2003; and Denis et al., 2016) or in the general population (MacLaren & Best, 2010; and Schluter et al., 2018). The remaining 4 tools screened for risk factors across a diverse range of health concerns including substance use and gambling in a primary health setting (Goodyear-Smith et al., 2004, 2009, 2013, 2021; and Elley et al.,. 2014) or for underlying behavioral traits in the general population (Chamberlain & Grant, 2018; Guo et al., 2017; and Rockloff and Dyer, 2006). These might be considered predictive tools rather than strictly screening.

### Therapeutic Intervention

Four broad typologies of therapeutic intervention for co-occurring gambling and substance use harms were identified in the literature (see Table 3): psychosocial intervention; pharmacological plus psychosocial intervention; on-referral to appropriate service; and non-invasive neurological intervention.

**Table 3:** Therapeutic interventions

Study	Intervention	Context	Sample	Measure	Gambling outcome	Substance outcome
Alho et al., 2022	Intranasal naloxone + MI (naloxone self-administered up to 4 x day over 12 weeks; 4 psychosocial interventions) [with placebo + MI control]	Individuals recruited to study with SOGS-R score ≥ 5	126 (62 naloxone + MI; 64 placebo + MI)	SOGS-r; G-SAS; VAS; PGSI; NODS; DSM-5; IDS9-SF; EUROHIS-8; AUDIT; MADRS; C-SSRC	No statistically significant difference in G-SAS between groups to week 14. No statistically significant difference between groups in improvements across all measures.	Improvements across measures but no statistically significant difference between groups
Cavicchioli et al., 2020	DBT-ST	Outpatient substance use clinic; secondary analysis of data that showed improvements in alcohol and substance use disorders.	186 (gambling disorder n = 6)	ASI; SCID- 5-PD; SPQ; DERS; AAQ-II;	Statistically significant improvements in gambling at end of treatment; improvements in emotion regulation; reduction in experiential avoidance	Earlier study showed significant and moderate to large improvements in CDA, severity of AUD, CO-SUDs and DER
Chan et al., 2018	RESTART program (ACT)	RESTART short residential program for ‘addiction’ (excluding illicit drug use)	86 (44 program; 42 control)	Health Consciousness Scale; Motivation to Change Scale; K10; Distress Disclosure Scale; Perceived Disturbance by Addiction; Gambling Self-efficacy Scale	Strengthened self-efficacy at 2 months; increased willingness to disclose distress at 2 months; decreased perceived interference by addiction at 2 months; increased motivation to change at post-treatment	Strengthened self-efficacy at 2 months; increased willingness to disclose distress at 2 months; decreased perceived interference by addiction at 2 months; increased motivation to change at post-treatment
de Castella et al., 2011	Referral to specialist gambling support service with 6-month follow-up	Gambling harms identified in mental health treatment seeking (with co-occurring substance use in 57% of sample)	Experiencing gambling harm (self) at screening (n = 50). Interview x 1 (n = 21) Interview x 2 (n = 15)	BAI; BDI-II; BIS-11; DAST; MAGS; Brief MAST; FPQOLI; SCL-90-R; SIS; BSI	Reduced severity of MAGS (not statistically significant)	Statistically significant reduction in Brief MAST (as well as non-substance use measures: BAI; BDI-II; and SIS)
Gay et al., 2017	rTMS; 1 x sham; 1 x active over 2 weeks)	Treatment seeking gambling disorder as identified by DSM IV-TR (GD) criteria	22	PG-YBOCS; craving VAS; active / sham treatment	Significant decrease in cue-induced craving (VAS) at 7 days; no significant difference in gambling behavior (PG-YBOCS) or in desire and control to gamble.	No outcomes recorded
Kim and Hodgins, 2018	CMAT	Working model only				
Lahti et al., 2010	Naltrexone + education and brief intervention over 19 weeks [pilot study – no control group]	Individuals recruited to study with SOGS-R and DSM-IV (for PG) score ≥ 5	39	PG-YBOCS; EQ-5D; BDI; AUDIT; self-assessment of gambling	Significant decrease in PG-YBOCS to week 4; significant increase in EQ-5D scores to week 16; significant decrease in BDI scores to week 16; gambling self-assessed as improved.	Small non-significant reduction in AUDIT scores
Martinotti et al., 2019	tDCS; daily for 5 days	Substance use and/or gambling treatment seeking at mental health service	34 (16 sham; 18 active)	Cue-induced craving; cocaine TLFB; BIS-11; HAM-A; HAM-D; Timeline Follow-back; VAS; Y-MRS	Statistically significant reduction at 5 days on VAS (as well as on BIS-11; HAM-A; HAM-D); statistically significant time x group effect in VAS craving	Statistically significant reduction at 5 days BIS-11. No results recorded for TLFB
Petry et al., 2016	Brief advice	Outpatient methadone clinics / psychosocial substance use treatment clinics	66	Days gambled; Dollars gambled; SOGS; TLFB; ASI	Reduced gambling; most rapid decline in gambling frequency; no significant reduction in ASI scores	No significant reduction in ASI scores
	MET + CBT		82		Reduced gambling; reduced dollars gambled and SOGS scores; results sustained to 24 months; no significant reduction in ASI scores	No significant reduction in ASI scores
	Education		69		Reduced gambling; no significant reduction in ASI scores	No significant reduction in ASI scores
Rowe et al., 2015	CBT	Substance use treatment	N/A	N/A	N/A. Inclusion in treatment manual for ATOD workers	N/A. Inclusion in treatment manual for ATOD workers
	MI					
Toneatto et al., 2002	CBT	Individuals recruited to study with DSM-IV score ≥ 5 Modalities combined for analysis	169	Lifetime and past month substance use; gambling abstinence / days abstinent; gambling treatment satisfaction; gambling treatment adherence	Improvements on gambling measures at end of 8-week treatment and retained to 12-months; decrease in illicit drug use post-treatment; no significant change in alcohol use post-treatment	Decrease in illicit drug use post-treatment; no significant change in alcohol use post-treatment
	Brief intervention					
	12-step					
	Individual solution-focused therapy					
Toneatto et al., 2009	Naltrexone + individual counselling (8 appointments over 12 weeks) [with placebo + counselling control]	Individuals recruited to study with gambling and substance use (assessed on DSM-IV criteria; TLFB; ADS)	52 (27 naltrexone + counselling; 25 placebo + counselling)	Alcohol Frequency; alcohol quantity / drinking days; gambling frequency; gambling expenditure / gambling days at post-treatment; 3 months, 6 months; 12 months	No significant difference between groups in improvements across measures.	Improvements across measures but no statistically significant difference between groups

Across 6 papers, 11 psychosocial interventions were identified (Cavicchioli et al., 2020; Chan et al., 2018; Kim & Hodgins, 2018; Petry et al., 2016; Rowe et al., 2015; and Toneatto et al., 2002). These papers looked at:

- interventions for gambling in substance use treatment seeking populations (n = 3);
- interventions for alcohol harms and gambling harms (not necessarily co-occurring) amongst a range of other behaviors (n = 1);
- the impact of substance use on gambling treatment outcomes (n = 1);
- the development of a working model for treatment, including treatment of co-occurring harms (n = 1).

One paper (Cavicchioli et al., 2020) comprised secondary analysis of an earlier study that had showed significant and moderate to large improvements in consecutive days of abstinence (CDA), severity of alcohol use disorder (AUD), severity of co-occurring substance use disorder (CO-SUD) and reduced difficulties in emotional regulation (DER) with dialectical behavioral therapy combined with skills training (DBT-ST). Other studies failed to measure substance use harms.

Despite not being validated, the component model of addiction (CMAT) has been included in the study, as it is the only tool that focuses on addressing vulnerability markers that appear to be common across gambling and substance use (Kim & Hodgins, 2018). It is a potential model of care that can inform the particular psychosocial interventions to be used in combination to address vulnerabilities.

In the 3 studies of pharmacological (opioid agonist) plus psychosocial interventions in co-occurring substance use and gambling harms, improvements were identified across measures including gambling and substance use across time (Alho et al., 2022; Toneatto et al., 2009; and Lahti et al., 2010). However, there was no statistically significant difference in improvements between groups administered active pharmacological plus psychosocial intervention and those administered placebo plus psychosocial intervention.

Only 1 study looked at on-referral (de Castella et al., 2011). In this study, those identified through screening as experiencing gambling harms (n = 50) were referred to a specialist gambling support service that provided individual counselling. They were also provided with information about self-exclusion and self-help groups. Only 15 of the 50 individuals identified for referral completed the follow-up interviews. Of these, 13 indicated that they had changed their gambling behavior, 8 had engaged in individual counselling (7 through the referral pathway) and 9 had attended a self-help group. Outcomes were measured at 6 months, with reduced severity scores across alcoholism (Brief MAST), anxiety (BAI), depression (BDI), gambling (MAGS), impulsivity (BIS-11), substance use (DAST), and suicide intent (SIS), and increased overall quality of life scores (FPQOLI). The reduction in gambling scores was not statistically significant and the median scores of gambling still placed participants within the Massachusetts gambling screen (MAGS) pathological gambling category. These results may be attributable to the small sample size. However, it is not unreasonable to conclude that the 35 individuals who were lost to follow up did not experience substantial reductions in gambling harm.

The literature identified 2 forms of non-invasive neurological intervention that have been validated in the context of co-occurring substance use and gambling harms (Gay et al., 2017; and Martinotti et al., 2019). Both of these studies found a statistically significant reduction in cue-induced craving on the visual analog scale (VAS). However, other measures of gambling behavior were either unaccounted for or showed no significant difference. Both studies had small sample sizes and measured only short-term effects.

## Discussion

Identifying individuals experiencing, or at risk of experiencing, gambling harms at an early stage has the potential to reduce harms. Screening for gambling harms takes on greater urgency where there are low levels of help-seeking behavior (Cunningham, 2005). Help-seeking, when it occurs, is typically crisis driven and in the context of severe harms such as bankruptcy, criminal behavior, or relationship breakdown (Evans & Delfabbro, 2005). The substance use treatment setting is ideally placed to provide screening for gambling (Himelhoch et al., 2015). Those self-selecting to attend an alcohol and other drug service already demonstrate motivation to change and so may be more amenable to additional interventions. While the rate of help-seeking amongst those experiencing substance use harms is still low, it is higher than the estimated 1 in 10 who seek treatment for the gambling harms they experience (Matheson et al., 2019; and Dschaak and Juntunen, 2018).

There is a wide range of screening tools available for identifying gambling harms in individuals seeking treatment for substance use harms. Across the dataset 11 gambling screening tools were identified that have been validated in substance use treatment-seeking populations. With the exception of the 17-item NODS and the 27-item GDSQ-P, these are short (9 items or less) and can easily be included as part of a broader intake process. Five gambling screening tools comprise 4 item or fewer and have been developed to rapidly assess gambling harms. Brief screening can take a clinician less than 2 min to complete and requires little interpretive skill (Himelhoch et al., 2015). Services might select an appropriate screening tool based on a balance of brevity, client and clinician acceptability, sensitivity and specificity. Screening can flag the need for further clinical assessment before development of a treatment or referral plan.

A search of the literature failed to identify any screening tools for substance use validated in a gambling harm treatment-seeking population. This suggests a gap in the research that may, in part, reflect the relative lack of available treatment and support services for gambling. Broad spectrum or multi-diagnostic tools that screen for a range of so-called addictive behaviors may prove helpful for assessing co-occurring substance use and gambling harms where an individual does not have a primary presentation of substance use harm but may be less useful in populations in which gambling or substance use harms are already established. There are a wide range of substance use screening tools that have been validated in the general population. Some of the most widely used tools include the Alcohol, Smoking and Substance Involvement Screening Test (ASSIST); the Alcohol Use Disorders Identification Test (AUDIT); Cut down, Annoyed, Guilty and Eye-opener (CAGE); and Drug Use Disorders Identification Test (DUDIT). These screening tools have not been included in this study as there were no papers identified in the dataset on validation of substance use screening tools in a gambling treatment setting. This does not imply that such tools would be unsuitable for use in the gambling treatment context, but it does suggest the need for additional research.

The literature identified four broad spectrum or multi-diagnostic tools that may be useful in screening for both gambling and substance use. Given time and resource demands, many services have limited capacity to undertake lengthy screening (Dowling et al., 2019). The length of multi-diagnostic screening tools may make them prohibitive in a clinical setting. However, in some cases this may be offset by the capacity to undertake one extensive screen as opposed to multiple brief screens.

Transdiagnostic tools that assess risk or identify underlying behavioral traits may have limited utility in a treatment setting, where risk is already established and where individuals may, on average, be expected to score highly on measures of traits such as impulsivity, regardless of whether or not they are experiencing co-occurring harms. In some cases, however, identifying behavioral patterns or traits can help to target interventions to individuals. If an individual is high on traits this may signal increased likelihood of them experiencing not only substance use and gambling but also other related co-morbidities. Further research on the validity of these tools in a clinical setting or in a treatment seeking population is needed to assess their utility in screening for co-occurring gambling and substance use harms.

There are a number of considerations that may apply when choosing the most appropriate screening tool for co-occurring harms. These include but are not necessarily limited to the treatment setting; the presence or otherwise of a primary diagnosis of substance use disorder or gambling disorder; the expertise of staff in undertaking and interpreting screening; the time available in a clinical setting; and the willingness and capacity of service users to engage with a potentially lengthy screening process. The options for gambling screening tools in a substance use setting are well-covered in the literature. At present, screening for co-occurring substance use and gambling in a non-substance use setting is limited to broad spectrum or multi-diagnostic tools. Validation of existing substance use screening tools in a gambling treatment setting is needed.

There is an established evidence base for a range of therapeutic interventions for substance use harms, including psychosocial interventions (Manuel et al., 2011), pharmacological interventions (O’Connor & Fiellin, 2000; and Amato et al., 2005), and non-invasive neurological interventions (Lupi et al., 2017). There is a similar, growing evidence base for the treatment of gambling harms (Toneatto & Millar, 2004). However, relatively few studies have looked at treatment options for co-occurring substance use and gambling harms. This study identified only 13 papers that had looked at the efficacy of therapeutic interventions for those experiencing such co-occurring harms, including one study that examines a model of care—the CMAT—that has not yet been integrated into practice (Kim & Hodgins, 2018).

There is evidence in the literature for the use of dialectical behavioral therapy (DBT) modified with the addition of skills training (ST) to reduce the severity of gambling in individuals seeking treatment for substance use. Combined cognitive behavioral therapy (CBT) and motivational enhancement therapy (MET) were found to reduce days gambled, dollars gambled and SOGS scores in individuals seeking treatment for substance use, with results sustained to 24 months. Both of these treatment modalities require a high level of sustained engagement from a population that is known to have poor treatment engagement and high rates of early treatment cessation (Abbott et al., 2017). Brief advice can be efficacious in achieving rapid declines.

Treatment outcomes for psychosocial interventions across co-occurring harms (i.e. outcomes for both substance use and gambling) are not well-recorded in the literature. A study of the pilot RESTART program, which utilises a form of acceptance and commitment therapy (ACT), offered some cautiously optimistic results to two months. However, the program specifically excluded those experiencing substance use harms, with the exception of tobacco and alcohol. Furthermore, results were not broken down by harms experienced, making it difficult to assess outcomes for the population of interest to this study. The study by Toneatto et al. found improvements in gambling measures sustained to 12 months, along with a reduction in illicit drug use at post-treatment and no reduction in alcohol use. Harm is not distinguished from use in this study and the combining of treatment modalities for the purpose of analysis means no conclusions about the efficacy of a specific treatment type can be drawn.

Studies of opioid agonist treatment (naloxone or naltrexone) combined with psychosocial interventions showed some improvements on gambling and substance use measures over time. However, studies that controlled between active opioid agonist treatment plus psychosocial intervention and placebo plus psychosocial intervention did not discern a statistically significant difference between outcomes. This suggests that psychosocial interventions may have been the critical factor in treatment outcomes. Opioid agonists may be appropriate in some treatment contexts but will be counter-indicated in others. For example, opioid agonists will precipitate withdrawal in patients using opiates including buprenorphine and methadone (Aboujaoude & Salame, 2016).

Based on a single study there is inadequate evidence to support on-referral at this time. However future studies could examine different referral and follow up protocols. On referral that involves close collaboration between ATOD and gambling support service providers is an area that could benefit from further research to understand treatment outcomes across both substance use and gambling measures.

While neurological interventions (rTMS and tDCS) showed some results on cue-induced craving (VAS) measures, there was no evidence of improvements across other gambling or substance use measures. The available studies had small sample sizes and measured only shortterm outcomes. Further research is required to illuminate any benefits of non-invasive neurological interventions for a broader population of those experiencing co-occurring gambling and substance use harms. Research might also consider the efficacy of rTMS or tDCS combined with longer term psychosocial interventions to achieve sustained outcomes across measures of gambling and substance use. At the same time, it should be recognized that there is low likelihood of rTMS and/or tDCS being easily integrated into current substance-use treatment settings.

## Conclusions

Developing best practice approaches to co-occurring gambling harms and substance use harms is an ongoing project that will need to adapt to an emerging body of evidence. This research has identified a range of potentially useful validated tools for clinicians in substance use treatment settings to screen for gambling harms. For workers in gambling treatment settings who are seeking validated tools to screen for co-occurring substance use harms, the literature provides less guidance. Substance use screening tools are widely available but have not been validated in a gambling treatment setting. Across both sectors and for clinicians in other healthcare settings (such as mental health or primary care) there are broad spectrum tools that can identify both gambling and substance use or identify underlying behavioral traits. However, these have various drawbacks including length, complexity and, in some cases, an inability to distinguish between different forms of harm.

The validated toolbox of therapeutic interventions for those experiencing co-occurring substance use and gambling harms is relatively sparse. Psychosocial interventions appear to offer the best outcomes on gambling measures for those experiencing co-occurring substance use harms. Further research is needed to establish the benefits of different combinations of treatment and treatment types in achieving reductions across both substance use and gambling harms, when these harms are experienced concurrently.

## References

[CR1] Abbott M, Bellringer M, Vandal AC, Hodgins DC, Battersby M, Rodda SN (2017). Effectiveness of problem gambling interventions in a service setting: A protocol for a pragmatic randomised controlled clinical trial. British Medical Journal Open.

[CR2] Aboujaoude E, Salame WO (2016). Naltrexone: A Pan-Addiction treatment?. Cns Drugs.

[CR4] Algren, M. H., Ekholm, O., Nielsen, L., Ersbøll, K., Bak, C., & Andersen, T., P (2018). Associations between perceived stress, socioeconomic status, and health-risk behaviour in deprived neighbourhoods in Denmark: A cross-sectional study. *Bmc Public Health*, *18*(250), 10.1186/s12889-018-5170-x. 10.1186/s12889-018-5170-x PMC5812195 29439681

[CR5] Alho H, Mäkelä N, Isotalo J, Toivonen L, Ollikainen J, Castrén S (2022). Intranasal as needed naloxone in the treatment of gambling disorder: A randomised controlled trial. Addictive Behaviors.

[CR6] Amato L, Davoli M, Perucci CA, Ferri M, Faggiano F, Mattick RP (2005). An overview of systematic reviews of the effectiveness of opiate maintenance therapies: Available evidence to inform clinical practice and research. Journal of Substance Abuse Treatment.

[CR3] ATODA (2022). ACT alcohol and other Drug Workforce Profile 2021: Qualifications, remuneration and wellbeing.

[CR7] Bramer WM, Giustini D, de Jonge GB, Holland L, Bekhuis T (2016). De-duplication of database search results for systematic reviews in EndNote. Journal of the Medical Library Association.

[CR8] Cavicchioli M, Ramella P, Vassena G, Simone G, Prudenziati F, Sirtori F (2020). Dialectical behaviour therapy skills training for the treatment of addictive behaviours among individuals with alcohol use disorder: The effect of emotion regulation and experiential avoidance. American Journal of Drug and Alcohol Abuse.

[CR9] Chan MLE, Cheung WTN, Yeung NYD, Kwok FPA, Wong HYR (2018). An evaluation study of the “RESTART” Program—Short-Term Residential Treatment for Addiction. International Journal of Mental Health and Addiction.

[CR70] Chamberlain, S. R., & Grant J. E. (2018). Initial validation of a transdiagnostic compulsivity questionnaire: The Cambridge–Chicago compulsivity trait scale. *CNS Spectrums, 23*(5), 340–346. 10.1017/S1092852918000810. 10.1017/S1092852918000810 PMC6124637 29730994

[CR10] Christo G, Jones SL, Haylett S, Stephenson GM, Lefever RMH, Lefever R (2003). The shorter PROMIS questionnaire: Further validation of a tool for simultaneous assessment of multiple addictive behaviours. Addictive Behaviors.

[CR11] Cowlishaw S, Merkouris S, Chapman A, Radermacher H (2014). Pathological and problem gambling in substance use treatment: A systematic review and meta-analysis. Journal of Substance Abuse Treatment.

[CR12] Cunningham JA (2005). Little use of treatment among problem gamblers. Psychiatric Services.

[CR13] Dale CF, Fontana VC, Martinez JA (2016). What’s your ‘vice?’: A combined approach to drugs and other addictive substances and activities. Addiction Research & Theory.

[CR14] De Castella A, Bolding P, Lee A, Cosic S, Kulkarni J (2011). Problem gambling in people presenting to a public mental health service.

[CR15] Denis C, Fatséas M, Beltran V, Serre F, Alexandre JM, Debrabant R (2016). Usefulness and validity of the modified addiction severity index: A focus on alcohol, drugs, tobacco, and gambling. Substance Abuse.

[CR16] Dowling NA, Cowlishaw S, Jackson AC, Merkouris SS, Francis KL, Christensen DR (2015). Prevalence of psychiatric co-morbidity in treatment-seeking problem gamblers: A systematic review and meta-analysis. Australian New Zealand Journal of Psychiatry.

[CR17] Dowling NA, Merkouris SS, Dias S, Rodda SN, Manning V, Youssef GJ (2019). The diagnostic accuracy of brief screening instruments for problem gambling: A systematic review and meta-analysis. Clinical Psychology Review.

[CR18] Dschaak ZA, Juntunen CL (2018). Stigma, substance use, and help-seeking attitudes among rural and urban individuals. Journal of Rural Mental Health.

[CR19] Eby, L. T., Mitchell, M., & Zimmerman, L. (2016). Work and family in times of crisis. In T. D. Allen, & L. T. Eby (Eds.), *The Oxford Handbook of Work and Family* (pp. 417–430). Oxford University Press. 10.1093/oxfordhb/9780199337538.013.32.

[CR20] Elley CR, Dawes D, Dawes M, Price M, Draper H, Goodyear-Smith F (2014). Screening for lifestyle and mental health risk factors in the waiting room: Feasibility study of the case-finding Health Assessment Tool. Canadian Family Physician.

[CR21] Evans L, Delfabbro PH (2005). Motivators for change and barriers to help-seeking in australian problem gamblers. Journal of Gambling Studies.

[CR22] Gay A, Boutet C, Sigaud T, Kamgoue A, Sevos J, Brunelin J (2017). A single session of repetitive transcranial magnetic stimulation of the prefrontal cortex reduces cue-induced craving in patients with gambling disorder. European Psychiatry.

[CR24] Goodyear-Smith F, Arroll B, Sullivan S, Elley R, Docherty B, Janes R (2004). Lifestyle screening: Development of an acceptable multi-item general practice tool. The New Zealand Medical Journal.

[CR26] Goodyear-Smith F, Warren J, Elley CR (2013). The eCHAT program to facilitate healthy changes in New Zealand primary care. Journal of the American Board of Family Medicine.

[CR25] Goodyear-Smith F, Darragh M, Warren J (2021). VeCHAT: A proof-of-concept study on screening and managing veterans’ mental health and wellbeing. Journal of Primary Health Care.

[CR71] Goodyear-Smith, F., Arroll, B., & Coupe, N. (2009). Asking for help is helpful: Validation of a brief lifestyle and mood assessment tool in primary health care. *The Annals of Family Medicine, 7*(3), 239–244. 10.1370/afm.962. 10.1370/afm.962 PMC2682962 19433841

[CR27] Grant JE, Chamberlain SR (2014). Impulsive action and impulsive choice across substance and behavioral addictions: Cause or consequence?. Addictive Behaviors.

[CR28] Grant JE, Chamberlain SR (2020). Gambling and substance use: Comorbidity and treatment implications. Progress in Neuro-Psychopharmacology and Biological Psychiatry.

[CR29] Guo K, Youssef GJ, Dawson A, Parkes L (2017). A psychometric validation study of the impulsive-compulsive Behaviours Checklist: A transdiagnostic tool for addictive and compulsive behaviours. Addictive Behaviors.

[CR30] Haddaway NR, Collins AM, Coughlin D, Kirk S (2015). The role of Google Scholar in evidence reviews and its applicability to grey literature searching. PloS One.

[CR31] Himelhoch SS, Miles-McLean H, Medoff DR, Kreyenbuhl J, Rugle L, Bailey-Kloch M (2015). Evaluation of brief screens for gambling disorder in the substance use treatment setting. American Journal of Addiction.

[CR32] Johansson A, Grant JE, Kim SW, Odlaug BL, Gunnar Götestam K (2009). Risk factors for problematic gambling: A critical literature review. Journal of Gambling Studies.

[CR33] Kim HS, Hodgins DC (2018). Component model of Addiction Treatment: A pragmatic transdiagnostic treatment model of behavioral and substance addictions. Frontiers in Psychiatry.

[CR34] Kim HS, Hodgins DC, Garcia X, Ritchie EV, Musani I, McGrath DS (2021). A systematic review of addiction substitution in recovery: Clinical lore or empirically-based?. Clinical Psychology Review.

[CR35] Kourgiantakis T, Ashcroft R, Mohamud F, Fearing G, Sanders J (2021). Family-focused practices in addictions: A scoping review. Journal of Social Work Practice in the Addictions.

[CR36] Lahti T, Halme JT, Pankakoski M, Sinclair D, Alho H (2010). Treatment of pathological gambling with naltrexone pharmacotherapy and brief intervention: A pilot study. Psychopharmacology Bulletin.

[CR37] Langham, E., Thorne, H., Browne, M., Donaldson, P., Rose, J., & Rockloff, M. (2016). Understanding gambling related harm: A proposed definition, conceptual framework, and taxonomy of harms. *Bmc Public Health*, *16*(80), 10.1186/s12889-016-2747-0. 10.1186/s12889-016-2747-0 PMC4728872 26818137

[CR38] Lorains FK, Cowlishaw S, Thomas SA (2011). Prevalence of comorbid disorders in problem and pathological gambling: Systematic review and meta-analysis of population surveys. Addiction.

[CR39] Loxley W, Toumbourou JW, Stockwell T, Haines B, Scott K, Godfrey C (2004). The prevention of substance use, risk and harm in Australia: A review of the evidence.

[CR40] Lupi M, Martinotti G, Santacroce R, Cinosi E, Carlucci M, Marini S (2017). Transcranial direct current stimulation in substance use disorders: A systematic review of scientific literature. The Journal of ECT.

[CR41] Maarefvand M, Mardaneh-Jobehdar M, Ghiabi M, Rafimanesh H, Mohammadi A, Morshedi Z (2019). Designing and evaluating the validity and reliability of the Persian Gambling Disorder Screening Questionnaire. Addiction & Health.

[CR42] MacLaren VV, Best LA (2010). Multiple addictive behaviors in young adults: Student norms for the shorter PROMIS questionnaire. Addictive Behaviors.

[CR43] Manning V, Dowling NA, Rodda SN, Cheetham A, Lubman DI (2020). An examination of clinician responses to problem gambling in community mental health services. Journal of Clinical Medicine.

[CR44] Manuel JK, Hagedorn HJ, Finney JW (2011). Implementing evidence-based psychosocial treatment in specialty substance use disorder care. Psychology of Addictive Behaviors.

[CR45] Martinotti G, Lupi M, Montemitro C, Miuli A, Di Natale C, Spano M (2019). Transcranial Direct Current Stimulation reduces craving in Substance Use Disorders: A Double-blind, placebo-controlled study. The Journal of ECT.

[CR46] Matheson, F. I., Hamilton-Wright, S., Kryszajtys, D. T., Wiese, J. L., Cadel, L., Ziegler, C., et al. (2019). The use of self-management strategies for problem gambling: A scoping review. *Bmc Public Health*, *19*(445), 10.1186/s12889-019-6755-8. 10.1186/s12889-019-6755-8 PMC6489359 31035978

[CR47] Milton AC, La Monica H, Dowling M, Yee H, Davenport T, Braunstein K (2020). Gambling and the role of resilience in an international online sample of current and ex-serving military personnel as compared to the general population. Journal of Gambling Studies.

[CR48] Munn, Z., Peters., M. D. J., Stern, C., Tufanaru, C., McArthur, A., & Aromataris, E. (2018). Systematic review or scoping review? Guidance for authors when choosing between a systematic or scoping review approach. *BMC Medical Research Methodology*, *18*(143), 10.1186/s12874-018-0611-x. 10.1186/s12874-018-0611-x PMC6245623 30453902

[CR49] Nelson KG, Oehlert ME (2008). Evaluation of a shortened South Oaks Gambling screen in veterans with addictions. Psychology of Addictive Behaviors.

[CR50] O’Connor PG, Fiellin DA (2000). Pharmacologic treatment of heroin-dependent patients. Annals of Internal Medicine.

[CR51] Peters MD, Godfrey CM, Khalil H, McInerney P, Parker D, Soares CB (2015). Guidance for conducting systematic scoping reviews. International Journal of Evidence-Based Healthcare.

[CR52] Peters, M. D., Godfrey, C., McInerney, P., Munn, Z., Tricco, A. C., & Khalil, H. (2020). Chapter 11: Scoping Reviews (2020 version). In Aromataris E, Munn Z (editors) *JBI Manual for Evidence Synthesis*. JBI. 10.46658/JBIMES-20-12.

[CR54] Petry NM (2003). Validity of a gambling scale for the addiction severity index. The Journal of Nervous and Mental Disease.

[CR53] Petry NM, Rash CJ, Alessi SM (2016). A randomized controlled trial of brief interventions for problem gambling in substance abuse treatment patients. Journal of Consulting and Clinical Psychology.

[CR55] Piasecki J, Waligora M, Dranseika V (2018). Google search as an additional source in systematic reviews. Science and Engineering Ethics.

[CR56] Potenza MN, Fiellin DA, Heninger GR, Rounsaville BJ, Mazure CM (2002). Gambling: An addictive behavior with health and primary care implications. Journal of General Internal Medicine.

[CR57] Rockloff MJ, Dyer V (2006). The four Es of problem gambling: A psychological measure of risk. Journal of Gambling Studies.

[CR58] Rowe C, White M, Long C, Roche A, Orr K (2015). Slots and shots: A Gambling Resource for AOD Workers.

[CR72] Schluter, M. G., Hodgins, D. C., Wolfe, J., & Wild, T. C. (2018). Can one simple questionnaire assess substance-related and behavioural addiction problems? Results of a proposed new screener for community epidemiology. *Addiction, 113*(8), 1528–1537. 10.1111/add.14166. 10.1111/add.14166 29357188

[CR60] Sherba RT, Martt NJ (2015). Overall gambling behaviors and gambling treatment needs among a statewide sample of drug treatment clients in Ohio. Journal of Gambling Studies.

[CR61] Sullivan S (2007). Don’t let an opportunity go by: Validation of the EIGHT gambling screen. International Journal of Mental Health and Addiction.

[CR62] Toneatto T, Millar G (2004). Assessing and treating problem gambling: Empirical status and promising trends. The Canadian Journal of Psychiatry.

[CR64] Toneatto T, Skinner W, Dragonetti R (2002). Patterns of substance use in treatment-seeking problem gamblers: Impact on treatment outcomes. Journal of Clinical Psychology.

[CR63] Toneatto T, Brands B, Selby P (2009). A randomized, double-blind, placebo-controlled trial of naltrexone in the treatment of concurrent alcohol use disorder and pathological gambling. American Journal of Addiction.

[CR65] Tricco AC, Lillie E, Zarin W, O’Brien KK, Colquhoun H, Levac D (2018). PRISMA extension for scoping reviews (PRISMA-ScR): Checklist and explanation. Annals of Internal Medicine.

[CR66] Volberg RA, Munck IM, Petry NM (2011). A quick and simple screening method for pathological and problem gamblers in addiction programs and practices. American Journal of Addiction.

[CR67] Wickwire EM, Burke RS, Brown SA, Parker JD, May RK (2008). Psychometric evaluation of the National Opinion Research Center DSM-IV screen for gambling problems (NODS). American Journal of Addiction.

[CR68] Wieczorek Ł, Dąbrowska K (2023). Unsatisfied treatment needs of people with comorbid alcohol/drug use and gambling disorder. Journal of Substance Use.

[CR69] Wolinski K, O’Neill M, Roche A, Freeman T, Donald A (2003). Workforce issues and the treatment of alcohol problems: A survey of managers of alcohol and drug treatment agencies.

